# Urban vectors of Chagas disease in the American continent: A systematic review of epidemiological surveys

**DOI:** 10.1371/journal.pntd.0011003

**Published:** 2022-12-14

**Authors:** Ana Laura Carbajal-de-la-Fuente, Paz Sánchez-Casaccia, Romina Valeria Piccinali, Yael Provecho, Liliana Salvá, Sergio Meli, Florencia Cano, Ricardo Hernández, Julieta Nattero

**Affiliations:** 1 Consejo Nacional de Investigaciones Científicas y Técnicas (CONICET), Buenos Aires, Argentina; 2 Centro Nacional de Diagnóstico e Investigación en Endemo-Epidemias (CeNDIE)-Administración Nacional de Laboratorios e Institutos de Salud "Dr. Carlos Malbrán" (ANLIS), Buenos Aires, Argentina; 3 Universidad de Buenos Aires, Facultad de Ciencias Exactas y Naturales, Departamento de Ecología, Genética y Evolución, Laboratorio de Eco-Epidemiología, Ciudad Universitaria—Ciudad Autónoma de Buenos Aires, Argentina; 4 CONICET—Universidad de Buenos Aires, Instituto de Ecología, Genética y Evolución (IEGEBA), Ciudad Universitaria—Ciudad Autónoma de Buenos Aires, Argentina; 5 Ministerio de Salud de la Nación, Dirección de Control de Enfermedades Transmitidas por Vectores, Ciudad Autónoma de Buenos Aires, Argentina; 6 Ministerio de Salud Pública de San Juan, Programa de Control de Enfermedades Transmitidas por Vectores, San Juan, Argentina; University of Texas at El Paso, UNITED STATES

## Abstract

**Background:**

Chagas is a complex and multidimensional socio-environmental health phenomenon, in which different components converge and interact. Historically, this disease was associated with insect vectors found in the rural environment. However, in the Americas, we are currently facing a new paradigm, in which different scenarios allow maintaining the vectorial transmission of the parasite through triatomine populations that either occasionally enter the dwellings or colonize urban environments.

**Methodology/Principal findings:**

Records of scientific reports available in the PubMed and LILACS search engines were retrieved, using three criteria according to the main triatomine genera of epidemiological importance and to the general scientific production on Chagas disease in urban contexts. Results showed that records on the occurrence of vectors in urban dwellings began to increase in the last three decades. Results also showed that the main species of triatomines collected inside dwellings (18 in total) belong mainly to the genera *Triatoma* and *Panstrongylus*, with most species (16/18, 88.8%) infected with the parasite, and that infestation of triatomine species occurs in all types of cities (small, medium and large, including megalopolises), from Argentina to the USA.

**Conclusions/Significance:**

Urban Chagas represents a new challenge that adds a different dimension to the problem of Chagas disease due to the particular characteristics of the lifestyle in urban agglomerates. The new scenario will require adaptations of the programs of control of vector to this shift from rural to urban settlements.

## Author summary

Chagas disease is caused by the parasite *Trypanosoma cruzi* and transmitted to humans through insects called triatomines, which feed on warm-blooded animals. Although this disease has been historically associated with the rural environment, in the last years, triatomines have been found either entering urban dwellings intrusively or colonizing urban environments of Latin American cities. In this study, we reviewed publications referring to urban Chagas that include the presence of the main vectors of this disease to find out how important the occurrence of triatomines in large cities is, which triatomine species involved are, whether or not these insects are infected with the parasite, and what the geographical distribution of the vectors and the parasite is. The results confirmed the existence of vectors in urban areas of Latin America and showed that records began to increase in the last three decades. Species of the genera *Triatoma* and *Panstrongylus* were the ones mainly recorded in the dwellings and the ones most infected with the parasite. Records of infected triatomine species have been reported from Argentina to the USA. This new scenario of Chagas disease raises the need to adapt the control of urban populations of the vectors found in these areas.

## Introduction

From a biomedical perspective, Chagas disease is caused by infection with the parasite *Trypanosoma cruzi* and affects mammals, including humans [1]. Transmission of *T*. *cruzi* can occur by multiple pathways: vector-borne, oral (food-borne), through blood/blood products, pregnant person-to-child (congenital or vertical), organ transplantation and laboratory accidents [2]. In the Americas, the most frequent route of transmission is the vectorial one, through the feces of insects known as triatomines, which are infected with the parasite [2,3]. Chagas disease is considered one of the most important human parasitoses on this continent. The latest estimates showed that approximately 6 million people are affected and that about 172,000 new infections occurred during 2019 [4].

Since the relationship found between the parasite, the vector, its domestic habitat and the disease by Carlos Chagas in 1909, the epidemiological, environmental, social, political and cultural scenarios of the disease have been modified, showing an increasing dynamic complexity [1,5]. In addition, during the last decades, these scenarios have also been modified by changes in policies at national and international levels, as well as by human migratory processes, which have enhanced the dispersion of the parasite and vectors through different geographical areas [5].

Due to the absence of a vaccine and an effective treatment for the chronic forms of the disease, the chemical control of vectors is the main tool used to reduce triatomine populations and therefore the incidence of the disease. Chemical control consists in spraying houses and neighboring areas (peridomestic areas) with residual chemical insecticides [6]. After the advent of pyrethroid insecticides in the 1980s, complete suppression of domestic triatomine populations was considered technically feasible [4]. Subsequently, in the 1990s, countries endemic for Chagas disease, together with the Pan American Health Organization-World Health Organization (PAHO-WHO), launched a series of intergovernmental initiatives for the control and surveillance of the disease. Over the years, this programs allowed effectively controlling the transmission of *T*. *cruzi* by blood transfusion and organ transplantation. However, *T*. *cruzi* can be transmitted by more than 150 species of vectors widely distributed throughout the Americas and, therefore, the complete interruption of vector transmission still seems difficult [4].

Triatomine-mediated transmission of *T*. *cruzi* has been recorded in several countries of South America and Central America, and incipiently recognized in areas of southern USA [5]. Currently, there are 157 described triatomine species (154 living species and three fossil species) [7], which differ both in their epidemiological relevance [8] and in aspects of their biology (habitats, food sources, life cycles, etc.). To facilitate vector control activities, triatomines have been classified according to biological and operational aspects [9–11]. Recently, a new classification has recently been proposed, which considers four hierarchical levels for the decision-making of control agencies: species (native or non-native), populations (wild or non-wild), outbreaks of infestation (natural, domestic or peri-domestic), and isolated insects (which can be solitary invaders of dwellings or part of a hidden infestation hotspot) [4,12].

For more than 70 years, control of triatomine populations has been focused on dwellings of rural areas [13]. However, we are currently facing a new paradigm in the continent, in which biomedical, epidemiological, social and cultural aspects are combined in a multidimensional way [1]. In this scenario, the vectorial transmission of *T*. *cruzi* is maintained through populations of triatomines that colonize dwellings not only of rural or semi-rural environments, but also of purely urban environments [5,14]. The proportion of the Latin American population living in large urban agglomerations has increased significantly from 41% in 1950 to 82% at present [15]. Simultaneously with the trends of population growth and urbanization, there is an increasing record of events linked to emerging infectious diseases where the presence of animals and insect vectors creates conditions for the spread and transmission of zoonoses [16,17]. Until a few decades ago, vector diseases were observed only when humans were exposed to forest or jungle environments; however, in the last years, these diseases have undergone a resounding change, emerging in urban areas. Driven by climate change, drastic modifications are taking place in the occurrence, distribution and frequency of insect vectors and mammals that are definitive or intermediate hosts of many important tropical diseases [16]. For instance, climate change has been linked to the geographical spread of *Phlebotomus* sandfly species, vectors of leishmaniasis, and *Aedes albopictus*, a carrier of Zika, dengue, and chikungunya virus [18]. Furthermore, there are climatic barriers to dengue transmission in South America. The former barrier in South Brazil has moved further south as a result of the increase in temperature according to a recent paper [19]. The spatial distribution of triatomines from Venezuela and Chile may be affected by global climate change [20,21]. This new paradigm of the epidemiology of vector-borne diseases emerging in urban areas, where most of the world’s human population lives, needs a better understanding and management of the phenomena involved [22].

Regarding the transmission of Chagas, several studies have documented the infestation of urban dwellings by vectors of epidemiological importance, such as *Triatoma infestans* in the cities of Arequipa (Peru) [23], San Juan (Argentina) [14] and Cochabamba (Bolivia) [24], and *Panstrongylus geniculatus* in Caracas (Venezuela) [25,26]. However, the occurrence of triatomines in urban areas ("urban triatomism") and, consequently, its relevance in the vectorial transmission of *T*. *cruzi*, possibly related to some specific regions of the American continent, have not yet been evaluated exhaustively and objectively on a continental scale. In order to fill this gap, we systematically reviewed the published evidence on this topic, specifically addressing the following questions: (i) How important is the occurrence of triatomines in large cities?; (ii) What are the triatomine species that infest homes or green spaces in urban areas?; (iii) Are these triatomines infected with *T*. *cruzi*? and, consequently, Is there a risk of vector transmission?; and (iv) What is the current geographical distribution of the vectors and the parasite? Finally, we discuss the strategies available for vector surveillance and control in densely populated areas, whose ecological and sociocultural features are different than those of rural areas.

## Materials and methods

Records from 1909 to 2021 of the scientific production available in the search engines PubMed and LILACS (Latin American and Caribbean Literature in Health Sciences) were retrieved. Studies comparing other search engines conclude that PubMed provides experienced searchers with tools and functionality that help improve recall and numerous options in order to improve precision [27,28]. PubMed offers ideal update frequency and includes online early articles. In addition to having free access, it remains an optimal tool in biomedical electronic research. Because many investigations are published in Spanish and Portuguese (the predominant languages in Latin America), we included the LILACS search engine. We searched for scientific articles published in English, Portuguese and Spanish, following four search criteria: three according to the triatomine genera with the greatest epidemiological importance, using the following combination of keywords: "Triatoma + urban", "Rhodnius + urban" and "Panstrongylus + urban"; and the combination "Chagas + urban" to determine the proportion of publications on the general scientific production of Chagas disease in an urban context. The review process only included documents that reported the occurrence of vectors in dwellings in urban areas or in green spaces of the city where triatomine colonies could have developed (e.g. squares, parks, etc with diverse urban vegetation and animals). The criterion of including green spaces was related to the maintenance of the urban cycle of *T*. *cruzi*. Due to the absence of an insecticide spraying protocol for urban areas, where homes are built close together, separated by a short distance (e.g. a street with which they are opposite), or located in buildings with numerous floors, the vector control in these areas presents numerous operational and logistical difficulties. In this study we follow the criteria recommended by the United Nations Organization to define and delimit the concept of city [29]. We included works that report data from urban agglomerates with a population of approximately 50,000–4 million or more inhabitants, grouped into the following categories: 50,000–99,999; 100,000–499,999; 500,000–999,999; 1 to 3.9 million; and 4 million or more [modified from 30]. In all cases, we confirmed that the number of inhabitants was included in each of the publications. Only a few articles (n = 4) did not contemplate population density and therefore were not included in the corresponding maps. The following items were also considered: 1) classification of the study (study design, whether or not the intervention considered entomological survey in urban dwellings, etc); (2) descriptive information, including (2.i) description of the collection (how, where and what was done), (2.ii) characteristics of the study (place, time, population, environments, vector species and their infection with *T*. *cruzi*, technique for the analysis of the infection, associated animals, location of the dwelling where the infestation occurred (intradomestic and/or peri-domestic), and (2.iii) classification of triatomines following [11] and [12]; and (3) quality of the study, including the quality of the descriptions (universe, sample size, possible sampling biases, etc).

Opinion articles, articles that did not present entomological searches in urban environments, articles that did not present research results with laboratory vector populations, and articles that were not experimental works performed with insects collected in urban areas (e.g. including morphological, molecular analysis studies, etc) were excluded and/or considered irrelevant, and used only for the introduction and/or discussion of this study. The inclusion/exclusion of documents was independently evaluated by ALCF, JN and PSC and discrepancies were resolved by consensus.

Data were extracted independently, using predefined data fields. Subsequently, the results of the data extraction were reviewed and the inconsistencies were resolved by re-revising the original documents. The distribution and number of species reported, the biomes where they were observed, and their infection with *T*. *cruzi* were mapped using the QGIS Girona 3.0 free access program (QGIS Development Team 2018). The database for compiling the bibliographic documents was gathered in a spreadsheet for further analysis.

## Results

**Fig 1:**
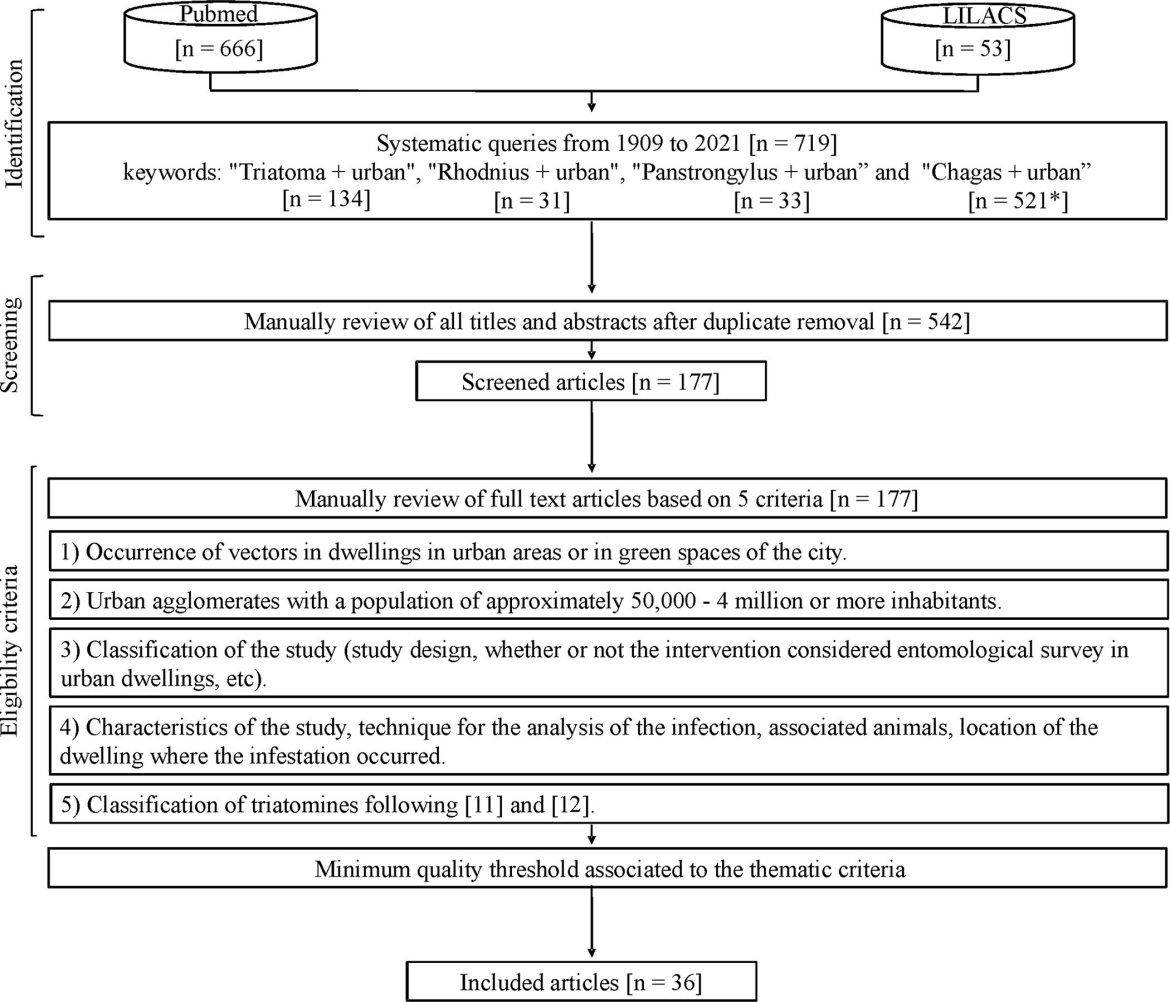
Flow diagram of the systematic review process. Scientific production available in the search engines PubMed (P) and LILACS (Latin American and Caribbean Literature in Health Sciences; L). * Registered value for Pubmed search.

Searches on the two engines used allowed retrieving a total of 719 documents. The largest number of documents was obtained using the combination of words "Chagas + urban" (72%, 521/719). However, this combination mostly reported documents that were excluded either because they were considered repetitions of works retrieved by one of the other criteria or because they did not meet the inclusion requirements. Most were studies related to the diagnosis and/or treatment of vertically transmitted Chagas disease. The PubMed search engine yielded the higher number of papers (n = 666), compared to LILACS (n = 53). However, only 36 articles satisfied the selected criteria (Fig 1, S1 Table).

**Fig 2:**
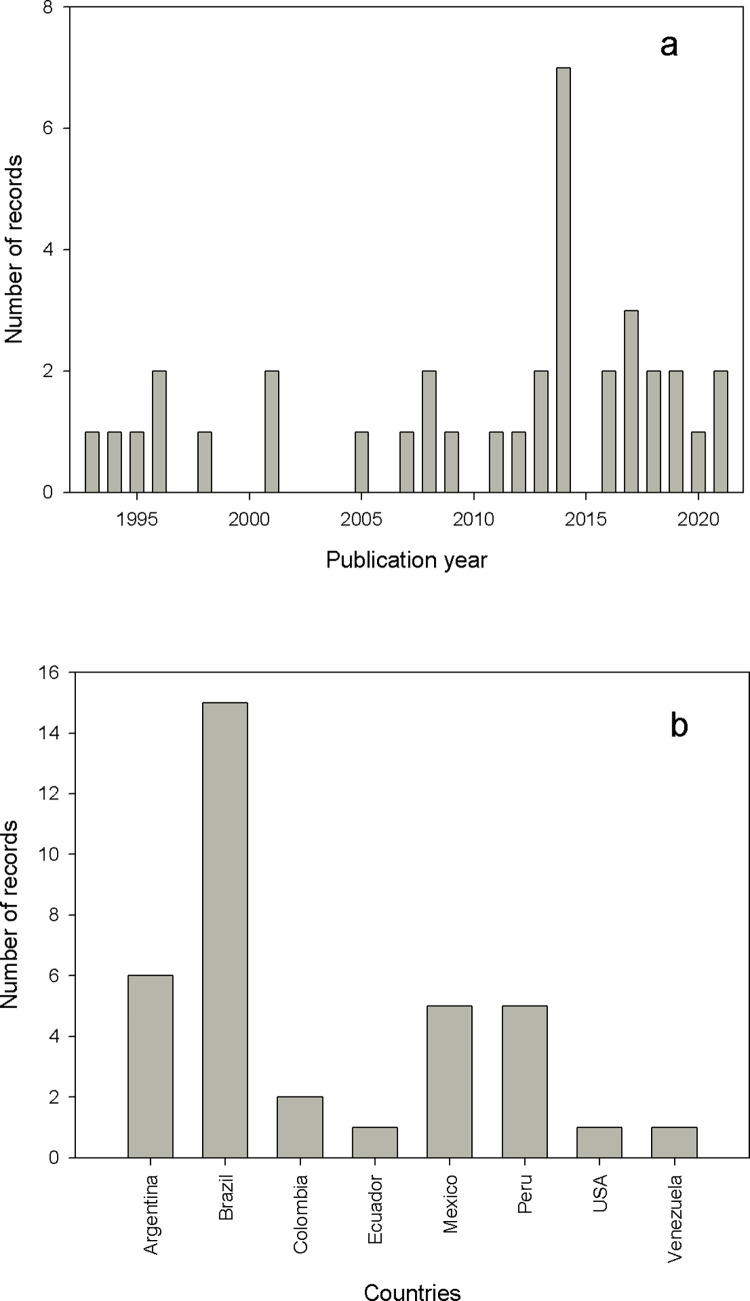
A) Number of records of vector occurrence in large urban agglomerates according to the years and B) countries where the vector was reported.

Although the search was focused on the period 1909–2021, the records that related the occurrence of vectors in dwellings in urban areas began to increase positively in the last three decades (Fig 2A). During the total period analyzed, the year 2014 showed the maximum number of reported works (n = 7). Four countries had at least five works reported: Brazil (n = 15), Argentina (n = 6), Mexico (n = 5) and Peru (n = 5), and the remaining four countries reported only one or two works (Fig 2B).

**Fig 3:**
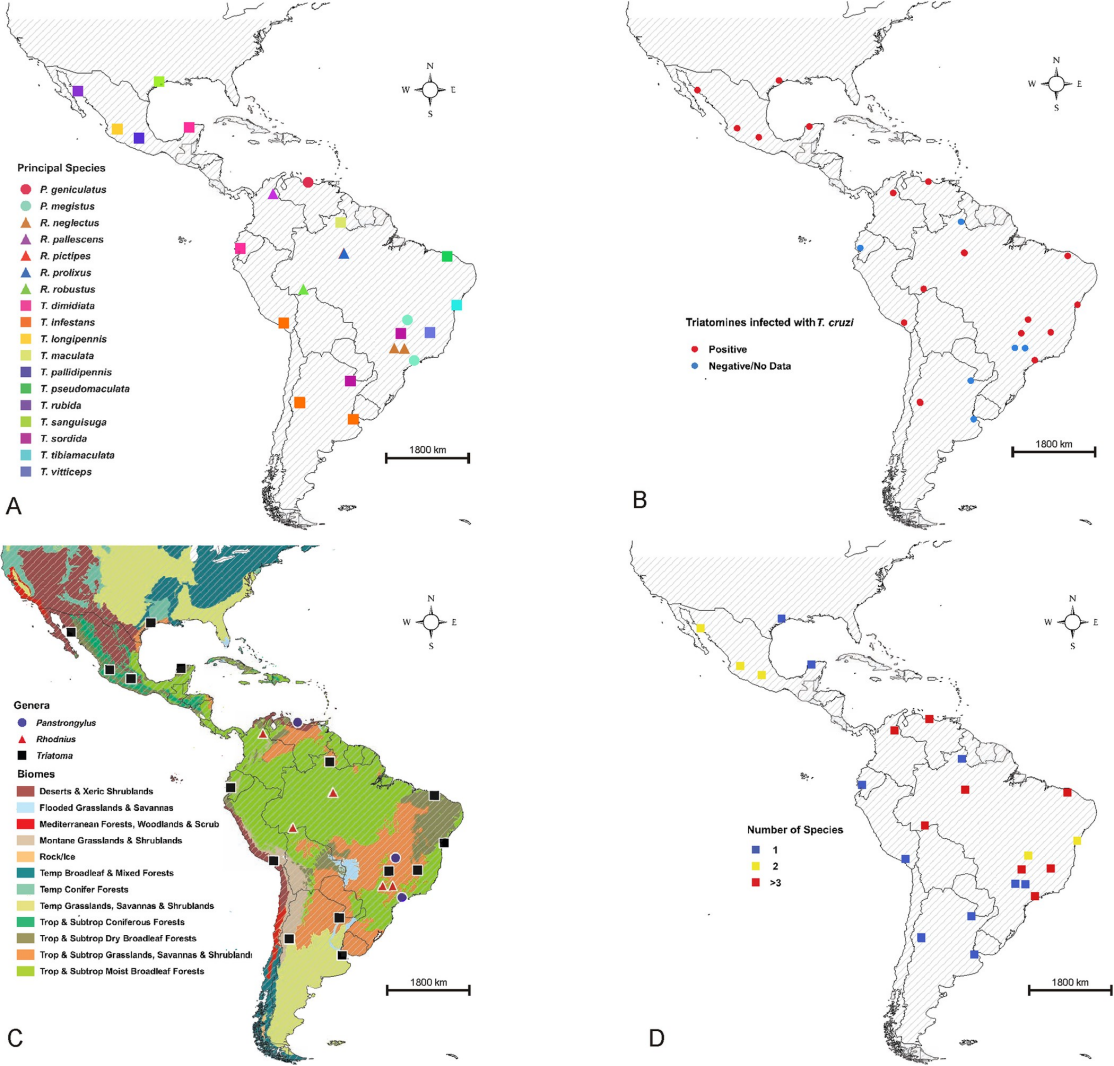
Geographical-ecological coverage of studies on occurrence of urban triatomines. A) Records of the main species that inhabit large urban agglomerates; B) Vectors infected with *Trypanosoma cruzi*; C) Biomes in which the three main genera of epidemiological importance have been reported; D) Records where several species have been reported. Maps were generated using the free open source geographic information system software QGIS Girona 3.0 free access program (QGIS Development Team 2018, https://www.qgis.org/es/site/); the shape files of the WWF Terrestrial Ecoregions of the World are freely available from https://www.worldwildlife.org/publications/terrestrial-ecoregions-of-the-world and the shape file of the Neotropical countries outlines are freely available at http://www.efrainmaps.es (Carlos Efraín Porto Tapiquén. Geografía, SIG y Cartografía Digital. Valencia, España, 2020).

The spatial and ecological coverage of the revised documents is represented in Fig 3A–3D. The spatial representation of the distribution of triatomines yielded 18 species distributed from Argentina to the USA, the vast majority infected with *T*. *cruzi* at least in one of the urban areas where each species was recorded (16/18) (Fig 3A and 3B). The largest number of species recorded was observed for the genus *Triatoma* (n = 11), followed by *Rhodnius* (n = 5) and *Panstrongylus* (n = 2), distributed in the geographical-ecological gradient represented in Fig 3C. The greatest species diversity was found in Brazil (n = 10), followed by Mexico (n = 4), Argentina and Colombia (n = 2), and Ecuador, USA, Peru and Venezuela (n = 1) (Fig 3A). Of the total documents reviewed, 47.2% (17/36) showed two species co-inhabiting the study area, while 27.8% (10/36) showed three or more species co-inhabiting the study area (Fig 3D).

**Fig 4:**
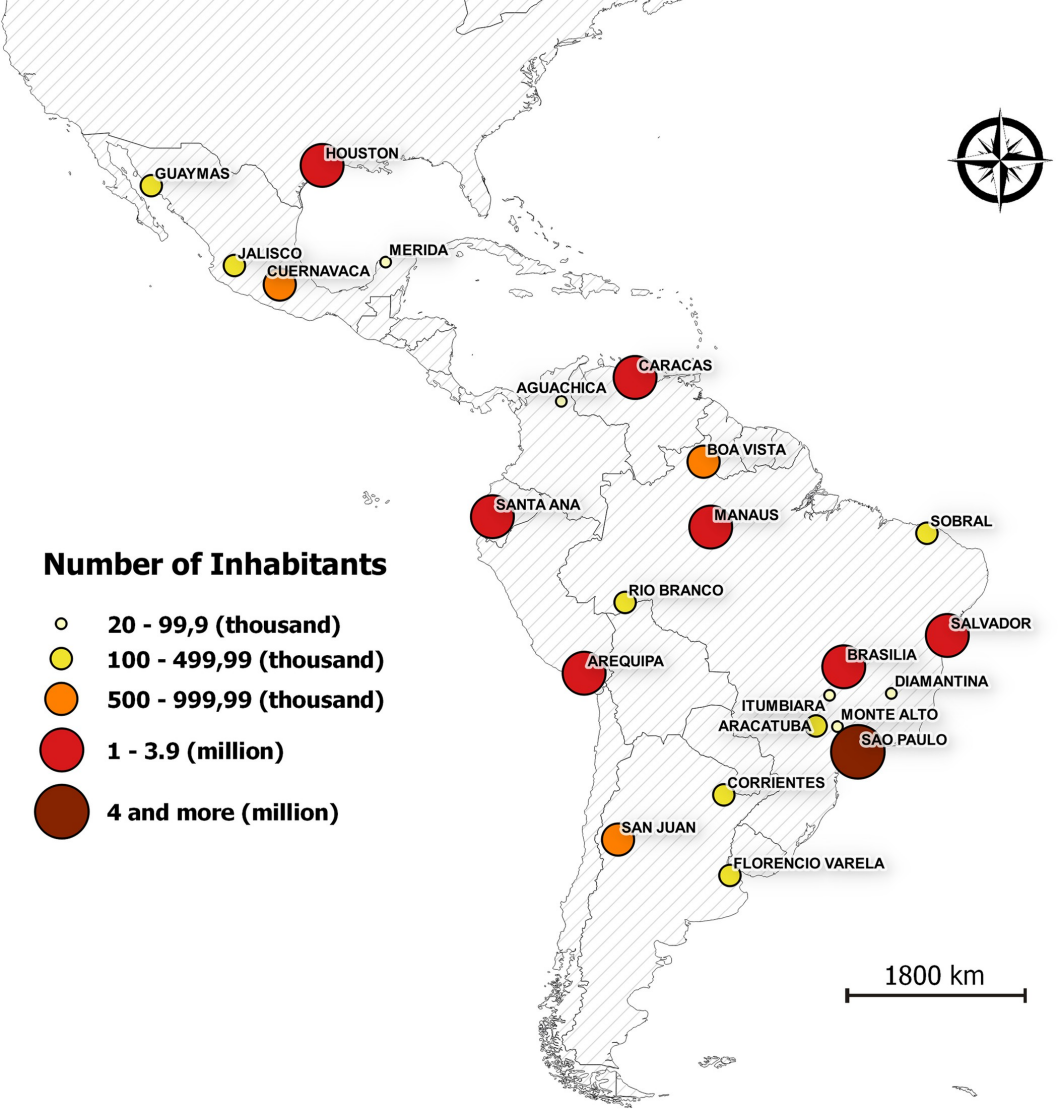
Urban agglomerates where urban vectors with their respective population density have been reported. Number of inhabitants according to *Comisión Económica para América Latina y el Caribe CEPAL* (2009) [30]. Map was generated using the free open source geographic information system software QGIS Girona 3.0 free access program (QGIS Development Team 2018, https://www.qgis.org/es/site/); the shape file of the Neotropical countries outlines are freely available at http://www.efrainmaps.es (Carlos Efraín Porto Tapiquén. Geografía, SIG y Cartografía Digital. Valencia, España, 2020).

The spatial coverage that associated the vector occurrence and *T*. *cruzi* infection with population density showed that this phenomenon occurs in cities with very heterogeneous population densities (Fig 4).

Table 1 summarizes the results on the occurrence of Chagas disease vectors and their prevalence of infection by *T*. *cruzi* [14,31–64]. The main species of triatomines collected inside dwellings belonged mainly to the genera *Triatoma* and *Panstrongylus*. *Triatoma infestans* (Argentina and Peru) and *Rhodnius prolixus* (Brazil) were reported indoors in urban dwellings. The former was found forming significantly abundant colonies, while the latter was found without signs of colonization. Both species showed high prevalence of *T*. *cruzi* infection (7.6% min—47.2% max for *T*. *infestans*; 71% for *R*. *prolixus*). Only *Triatoma sanguisuga* (found in the USA) and *Triatoma sordida* (found in Argentina -where it has also been recently called *Triatoma rosai*- and Brazil) were collected in association with the vegetation of green spaces. The species *Rhodnius neglectus* (Brazil) and *R*. *pallescens* (Colombia) were found colonizing palm trees in green spaces and were associated with the invasion of nearby dwellings. In Colombia, the occurrence of *R*. *pallescens* infected with *T*. *cruzi* was associated with an acute Chagas outbreak caused by oral transmission in 2010.

**Table 1:** Chagas disease vector occurrence in urban contexts: main triatomine species reported with greater abundance in the revised documents, intradomiciliary infestation and bug infection with *Trypanosoma cruzi*.

Vector	Country, (Province)	Evidence	Classification a/b	Reference
*P*. *geniculatus*	Colombia/ Venezuela	Infestation inside dwellings. Insects with a high prevalence of infection with *T*. *cruzi* (56% - 75%).	Sylvatic with domestic intrusion and with potential for domestication/ native	[25,31]
*P*. *megistus*	Brazil	Reports in the urban area of the cities of São Paulo and Brasilia from infested dwellings. Household infestation rate of 16%. Low prevalence of vector infection (3%).	nc/ native	[32–34]
*R*. *neglectus*	Brazil	Colonization of palm trees also inhabited by rodents, birds and marsupials and invasion of urban dwellings in cities in the state of São Paulo. No information about *T*. *cruzi* infection.	nc/ native	[35,36]
*R*. *pallescens*	Colombia	Association of the vector with the wild cycle of *T*. *cruzi* and oral transmission of Chagas disease. Found in palm trees located near a dwelling. High prevalence of vector infection (54%).	Sylvatic (palm tree) with some domestic intrusion/ native	[37]
*R*. *pictipes*	Brazil (Manaus)	Bioecological aspects of triatomines and marsupials as wild *T*. *cruzi* reservoirs in urban areas with occurrence of the vector inside dwellings, without colonization. Wide range of prevalence of vector infection (6%-41%).	nc/ native	[38,39]
*R*. *prolixus* ^***~***^	Brazil (Manaus)	Occurrence of the vector in urban dwellings, but without signs of colonization. Very high prevalence of vector infection (71%).	Domiciliated and sylvatic populations/ native	[40]
*R*. *robustus*	Brazil (Acre)	Occurrence of the vector in homes of a residential complex in Rio Branco. With intrusion, without domiciliation. High prevalence of vector infection (25%).	nc/ native	[41]
*T*. *dimidiata*	Mexico/ Ecuador	Report of the vector in urban dwellings in the cities of Mérida (Mexico). Marked endemicity in neighborhoods of Guayaquil (Ecuador). High prevalence of vector infection in Mérida (48%). Prevalence of infection not reported for Ecuador.	Not domiciliated/ native (Mexico;/ nc/ not native (Ecuador)	[42–43]
*T*. *infestans*	Argentina^+^ (San Juan)/ Peru (Arequipa)	Significant colonization of urban dwellings with varied infestation rate (7%-33%). Broad range of prevalence of vector infection (8%-47%). In Argentina, the only reports of acute Chagas by vector transmission for the country, located in the metropolitan area of San Juan city during 2016–2020.	Domestic/ native	[14,44–52]
*T*. *longipennis*	Mexico (Jalisco)	Occurrence of triatomines in dwellings, with an infestation rate of 18%. Prevalence of vector infection of 15%.	Peridomestic with domestic intrusion/ native	[53]
*T*. *maculata*	Brazil (Roraima)	Report of the vector in homes located in a residential complex in the city of Boa Vista. Vector without *T*. *cruzi* infection.	nc/ native	[54]
*T*. *pallidipennis*	Mexico (Morelos)	Infestation in urban dwellings in the city of Cuernavaca, with very high prevalence of vector infection (50% - 88%).	nc/ native	[55,56]
*T*. *pseudomaculata*	Brazil (Ceará)	Occurrence of the vector inside dwellings in the urban area of Sobral and other districts of the state. Variable prevalence of the vector (1%-18%). Urban hotspots revealed through community-based surveillance.	nc/ native	[57,58]
*T*. *rubida*	Mexico (Sonora)	Report in urban dwellings in Guaymas, with an infestation rate of 63% and very high prevalence of infection (90%).	nc/ native	[59]
*T*. *sanguisuga*	Southern USA (Texas)	First record of the vector in green spaces of the Houston urban area. Insects infected with *T*. *cruzi*.	Essentially sylvatic, with adults visiting human dwellings/ native	[60]
*T*. *sordida*	Argentina (Corrientes province)/ Brazil (Goiás)	In Corrientes (Argentina), urban infestation associated with pigeon nests in green spaces of the city, with evidence of intrusion to nearby homes. Insects infected with *T*. *cruzi*. In Itumbiara (Goiás), the vector colonizes domestic and peridomestic ecotopes, with a low prevalence of infection (5.5%).	Sylvatic with an advanced process of adaptation to human habitat/ native	[61–62]
*T*. *tibiamaculata*	Brazil (Bahía)	Occurrence of the vector in urban dwellings in San Salvador de Bahía, with an infestation rate of 48%. High prevalence of vector infection (54%).	nc/ native	[63]
*T*. *vitticeps*	Brazil (Minas Gerais)	Occurrence of the vector in the urban area of Diamantina. High prevalence of vector infection (19%).	nc/ native	[64]

a/b Triatomine classification criteria following a: (a) Waleckx et al. (2015) [11] and (b) Abad-Franch (2016) [12]; nc: not classified.

***^~^*** The occurrence of *R*. *prolixus* in houses from northern Brazil was challenged by Monteiro et al. [65] who stated that those records belong in fact to *R*. *robustus*.

+ Occurrence of *T*. *infestans* in urban dwellings in the town of Florencio Varela (province of Buenos Aires), in an area considered non-endemic for the vector.

The most frequently reported diagnostic technique used to determine *T*. *cruzi* infection in triatomines was observation of feces under the light microscope (21/36). Determination of infection by polymerase chain reaction (PCR) or direct immunoassay (ELISA) was rarely used (3/36). A notable proportion of documents did not report whether they had made a diagnosis of infection (11/36).

## Discussion

The complete review of the documents considered in this work allowed establishing a basis about the occurrence of triatomines in urban areas, the infestation of dwellings and green spaces, the circulation of *T*. *cruzi* in urban areas, and the size of the urban agglomerates where these events occur. Our results show that the urban infestation in the Americas is associated with 18 species of triatomines with varying degrees of adaptation to urban areas. The diagnosis of *T*. *cruzi* infection revealed that the parasite is transmitted by 17 different species of triatomines. The results also showed that the spatial and ecological coverage of triatomine infestation and parasite infection extends across the continent from Argentina to southern USA and that urban infestation has been increasing from the 1990s to the present. This phenomenon has become more prominent and reported more frequently over the past three decades. An important point to highlight is that at least 18 species of triatomines belonging to the genera *Panstrongylus*, *Rhodnius* and *Triatoma* occur in urban areas. The results of a study that modeled the geo-spatial distributions (including rural and urban areas) of the thirty most commonly reported triatomine species and putative vectors of *T*. *cruzi* partially coincide with our results, showing 14 of our 18 species reported in urban areas [66]. Other species such as *R*. *montenegrensis*, *R*. *nasutus*, *T*. *barbieri*, *T*. *brasiliensis*, *T*. *nigromaculata* and *T*. *recurva* have been found sharing ecotopes with some of the 18 species mentioned above (results not shown).

From a public health perspective, some triatomines are more relevant than others, a fact that depends primarily on how they interact with humans. The best-known classifications to date distinguish "primary" from "secondary" vectors or "domestic" from "wild" triatomines, occasionally with other subsets such as "candidate " or "intrusive" vectors versus "domiciliary" or "domestic" vectors (e.g., [67,68]). A review of triatomine species (or populations, depending on the region) classified them into at least 15 categories and subcategories ranging from "domestic" or "domesticated" to "essentially wild" (Table II of [11]). Since, operationally, some insects are easier to control than others, Zimmerer et al. [17] proposed a simple but robust classification that brings together biological and operational aspects so that vector control is feasible. In the documents analyzed, we found a great variability in the population density of urban spaces, ranging from 45–50 thousand to 14 million inhabitants, and a heterogeneity of vectors with different epidemiological importance (e.g. *T*. *infestans*, *P*. *geniculatus*, *R*. *neglectus*) inhabiting those spaces. *Triatoma infestans* is considered one of the most epidemiologically important vectors in South America. In the cities of San Juan (Argentina) and Arequipa (Peru), this species is considered native and non-native respectively [11], develops large colonies in urban dwellings, and has high prevalences of infection by *T*. *cruzi* [14,69]. Performing entomological surveillance and vector control in these scenarios represents a complex problem and a great challenge since some of these urban agglomerates reach 1 million inhabitants with a reality completely different from that of rural areas. However, this phenomenon has been little addressed and overlooked by vector control agencies [14].

Given the high density of people living in close proximity to domestic and peridomestic animals, urbanization can create conditions for the spread of vector transmission of diseases [70]. As a consequence, the urban environment implies a strong, novel and atypical challenge for control programs. Protocols and methodologies for vector control and evaluation, which were standardized in the 1960s, established the use of manual and/or automatic pumps for the application of insecticide [71,72]. In rural areas with an infestation rate <5%, the insecticide is applied following the selective methodology, whereas in those with an infestation rate >5%, the insecticide is applied following the attack methodology [73]. However, in the new scenario of urban infestation (e.g. with *T*. *infestans*), which is opposed to the rural one in architectural, socio-demographic, cultural and environmental terms, when finding an infested urban dwelling, control programs are frequently faced with questions such as: i) Should the insecticide be sprayed only in the infested dwelling, in the adjoining dwellings or in all the dwellings that make up the block?, ii) How to act if the infested home is located in a vertical building where there are several floors and apartments per floor?, iii) How to act in surrounding homes/buildings that are across the street: must they be included within the protocol to be evaluated and sprayed with the insecticide as well?, iv) How far should a vector control program be extended within the scope of a city that has more than 600,000 homes and scarce human and financial resources? and v) If we increase the scale of work, is it necessary to cover all the houses in the city? How much time and resources would this take? Questions such as these have led research groups and vector control programs at the national and provincial levels in both Argentina and Peru to unify efforts [69,74], with the aim to find answers that contribute to entomological surveillance and control according to the realities of each place. The unprecedented experience currently being developed in Argentina involves both national referents of the National Ministry of Health and international referents from Brazil and Uruguay, members of the technical teams of vector control agencies of the provinces affected (Catamarca, La Rioja, San Juan, Mendoza and San Luis) and a group of scientists who have been developing their research projects in urban Chagas [74].

The study was limited to a bibliographic search for a period greater than a century (1909–2021), but it did not consider data that could be obtained from direct sources like National or Provincial Vector Control Programs from countries with urban Chagas cases. This was due to the lack of shared information about the topic and the difficulties to request vector information among countries. The implementation of a public database collecting details on the vectors and circulation of *T*. *cruzi* infection in each country would be an interesting tool to allow accurate and up-to-date data for both rural and urban areas. Starting in the 1990s, Chagas disease-endemic countries and the Pan American Health Organization-World Health Organization (PAHO-WHO) launched a series of multinational initiatives for Chagas disease control-surveillance. An overview of the initiatives’ aims, achievements, and challenges can be found in Rojas de Arias et al. 2021 [4]. With an emphasis on the decentralization of the health sector in Latin America, the authors explore positive and negative aspects of the current structure of control programs. The advantages of incorporating a database with accessible information on vectors are not mentioned, though.

Numerous studies approach the problem of triatomine infestation and its infection by *T*. *cruzi* in peri or suburban areas [75–77]. However, the urban area concept is controversial since there are no unified criteria for its characterization. The definition of “rural” and “urban” differs between countries and regions. For example, there are countries that use the minimum population size to define an area as urban, but this number can be highly variable (from 200 inhabitants in Denmark; 2,000 in Argentina; 5,000 in India; 50,000 in Japan, or even 100,000 in China). Some countries do not use a definition based on population size but delimit urban areas according to administrative units. Other countries use sectorial employment or the availability of infrastructure and services to determine whether settlements should be classified as urban or rural. For this reason, the United Nations Organization [29] recommends a broader perspective to characterize the urban area. It postulates that cities are those spaces that have a population of at least 50,000 inhabitants (more than 1,500 inhabitants per square kilometer). Due to this complexity and following this criterion, only urban areas (and not peri-urban areas) were included in this research.

In Peru, an important collaborative work has been carried out for approximately two decades between local and foreign academic institutions, government authorities at different jurisdictional levels, and the Ministry of Health. Their interest focuses on the ecoepidemiology and control of Chagas disease in the city of Arequipa [51]. Numerous studies have been carried out in this city to advance the understanding of infestation by *T*. *infestans* and infection by *T*. *cruzi* in the urban context. It is already known that it is hindered by roads between city blocks, making it unlikely that the vector facilitates *T*. *cruzi* block-to-block migration [78]. Additionally, another study showed the number of domestic animals in and around each house. The results suggest that *T*. *cruzi* dispersal within a block occurs regularly and that occasional long-range dispersal events allow the establishment of new *T*. *cruzi* populations in distant blocks. Movement of domestic animals may be the primary mechanism of inter-block and inter-district *T*. *cruzi* dispersal [79].

Since the discovery of Chagas disease in 1909, abundant scientific evidence has been gathered, and research initially linked to vectors has played an important role in the history of Chagas disease control. This has allowed a decrease in vector and transfusion transmission as well as in the interruption of *T*. *cruzi* transmission in some areas of the continent. However, urban Chagas represents a new challenge that adds a different edge to the problem of Chagas disease due to the particular characteristics of urban agglomerates. Insecticide resistance is another difficult problem that may develop in an urban setting because it renders the most popular control methods ineffective. Triatomines may be subjected to insecticides used to control other urban insects in residential areas (i.e. cockroaches, mosquitoes, flies, ticks and others). In light of this novel situation, the insecticide resistance of urban triatomines is highly relevant and should be the focus of systematized monitoring by the responsible authorities. Over the years the WHO has recommended the use of an integrated approach to vector control, involving both chemical and nonchemical methods, and environmental management [80]. Integrated Vector Management (IVM) implies the simultaneous control of multiple diseases transmitted by different vector species in a certain area, or one tool controlling several Vector Borne Diseases transmitted by the same vector [81,82]. Currently, a new strategy has been launched in the Americas to reinforce the IVM approach and make it accessible to all-region vector control programs [83]. However, due to operational challenges that prevent the complete integration of IVM into program routines in most of the region’s countries, development has been gradual [83].

For all the above we believe that the most feasible proposal for the control of urban Chagas would be an integrative approach that considers the multidimensionality of the problem, determining the thresholds of infestation and infection by *T*. *cruzi*, diagnosing pregnant person and children, stimulating community participation through Information, Education and Communication activities, and considering spontaneous complaints by the population [1,12] adapted to the particularities of the lifestyle in big cities.

## Supporting information

S1 Table Documents that satisfied the selected criteria. (XLSX)
