# Correction: Urban vectors of Chagas disease in the American continent: A systematic review of epidemiological surveys

**DOI:** 10.1371/journal.pntd.0011384

**Published:** 2023-06-01

**Authors:** Ana Laura Carbajal-de-la-Fuente, Paz Sánchez-Casaccia, Romina Valeria Piccinali, Yael Provecho, Liliana Salvá, Sergio Meli, Florencia Cano, Ricardo Hernández, Julieta Nattero

The Funding statement is incomplete. The correct Funding statement reads:

This study was partially funded by Argentina’s National Promotion Agency for Science and Technology (PICTs 2018–3200 to ALCF and PICT 2018–1545). The funders had no role in study design, data collection and analysis, decision to publish, or preparation of the manuscript.

## References

[1] Carbajal-de-la-Fuente AL, Sánchez-Casaccia P, Piccinali RV, Provecho Y, Salvá L, Meli S, et al. (2022) Urban vectors of Chagas disease in the American continent: A systematic review of epidemiological surveys. PLoS Negl Trop Dis 16(12): e0011003. doi: 10.1371/journal.pntd.0011003 36516183 PMC9797073

